# Lipid Structure Determines the Differential Impact of Single Metal Additions and Binary Mixtures of Manganese, Calcium and Magnesium on Membrane Fluidity and Liposome Size

**DOI:** 10.3390/ijms24021066

**Published:** 2023-01-05

**Authors:** Kevin Sule, Max Anikovskiy, Elmar J. Prenner

**Affiliations:** 1 Department of Biological Sciences, University of Calgary, Calgary, AB T2N 1N4, Canada; 2 Department of Chemistry, Nanoscience Program, University of Calgary, Calgary, AB T2N 1N4, Canada

**Keywords:** lipids, membrane fluidity, metal mixtures, metal–lipid interactions, model membranes

## Abstract

Unilamellar vesicles of the biologically relevant lipids phosphatidic acid (PA) and phosphatidylserine (PS) with fully saturated (DM-) or partly unsaturated (PO-) acyl side chains were exposed to Ca, Mn and Mg in single metal additions; in equimolar mixtures or by sequential additions of one metal at a time. Laurdan generalized polarization measured the membrane fluidity, while dynamic light scattering reported liposome size changes complemented by zeta potential. All metals induced membrane rigidity and increased liposome sizes across all systems. Mn had the strongest effect overall, but Mg was comparable for DMPS. Lipid side chain architecture was important as GP values for binary mixtures were higher than expected from the sum of values for single additions added to POPS but smaller for DMPS. Sequential additions were predominantly different for Ca:Mg mixtures. Mn induced the strongest increase of liposome size in saturated lipids whereas Ca effects dominated unsaturated matrices. Binary additions induced larger sizes than the sum of single additions for POPS, but much lower changes in DMPA. The order of addition was relevant for PS systems. Thus, lipid structure determines metal effects, but their impact is modulated by other ions. Thus, metal effects may differ with the local lipid architecture and metal concentrations within cells.

## 1. Introduction

Essential metals, required for proper growth and development, are found within the extra- and intracellular pool of living organisms [1]. Manganese (Mn), calcium (Ca), and magnesium (Mg) are essential trace metals necessary for proper cell function [2]. Manganese is highly abundant in the earth’s crust; hence it is prevalent in plants and soil and is a nutritional requirement in our diet. The tolerable concentrations of Mn are below 50 µM, while higher amount can lead to metal toxicity [3] and chronic exposure has resulted in a neurological disorder known as Manganism, with similar symptoms to Parkinson’s disease [4]. Calcium is naturally found in teeth and bones and is an important secondary messenger in cells [5]. At the cellular level, calcium is found at mM levels. This ubiquitous element is capable of binding to many proteins thus acting as a cofactor [5,6]. However, cellular calcium levels are tightly controlled through sequestration by specialized proteins. Similarly, magnesium is an important cofactor in many metabolic enzymes [7] but has a much weaker binding affinity towards proteins [8]. Both Ca and Mg play a vital role in the structure and function of nucleic acids, necessary for a proper genomic machinery [9]. Moreover, these three metals can substitute each other as cofactors when one metal is scarce [10].

Given the widespread use of metals in daily human activities, toxic heavy metals have the propensity to bioaccumulate in living systems [11]. Out of the three metals examined in this work, only Mn has the potential to bioaccumulate, whilst Ca and Mg are ubiquitous in the body. Most studies on Mn bioaccumulation have been known from fish and plant models, whereby the uptake and buildup of excess Mn is a result of by products being released into the environment due to industrial processes such as welding and gasoline additives [12,13]. At the cellular level, bioaccumulation results in organelle dysfunction, such as lysosomal/vacuolar swelling and oxidative damage to the mitochondria [14,15]. Hence, these divalent cations support a plethora of biomolecular interactions at the cellular level.

Lipids are targets for divalent cations due to electrostatic interactions with specific classes [16,17]. Divalent cations may cause increased membrane rigidity [18], aggregation [19], phase separation [20] and enhanced membrane permeability [21] in both model and isolated biological membranes. Electrostatic interactions make anionic glycerophospholipids common metal targets.

In eukaryotic membranes, phosphatidic acid (PA) and phosphatidylserine (PS) are bioactive lipids that have important signaling functions necessary for proper membrane function and cellular homeostasis [22,23]. These lipids were selected for the current work due to their biological significance. Biomimetic lipid systems are useful models for monitoring lipid specific changes in membrane properties induced by divalent cations as they allow for accurate control over lipid composition, in contrast to isolated natural membranes [24].

PAs act as intermediates in the biosynthetic route of other phospholipids. They are present at 1% of the phospholipid content in eukaryotic membranes. These lipids are linked to signal transduction, cytoskeletal rearrangement, trafficking and secretion [25], and their content is highly regulated. PAs have the simplest structure of any phospholipid with a phosphate headgroup.

PS lipids contain a serine head group connected to the phosphate linker and are mainly localized in the inner leaflet of the plasma membrane. Similar to PA, PS serves signaling functions such as apoptosis promoting the blood coagulation cascade [26]. Considering the electrostatic attraction of divalent cations, it is important to understand the impact of divalent cations on the membrane properties of PA and PS, particularly their phase state.

The interactions of Ca, Mn and Mg with anionic lipid membranes have been investigated and Ca has been identified as a fusogenic agent by promoting the formation of the fusion stalk and by activating fusogenic enzymes [8,19,27]. Furthermore, Ca, Mn and Mg induce phase separation in monolayers composed of phosphatidylcholine, and PS has been shown by atomic force and Brewster angle microscopy [28].

The main objective of this work is to probe the interactions of Ca, Mn and Mg in a 1:1 metal mixture with PA and PS membranes using fluorescence spectroscopy, dynamic light scattering, and zeta potential. The significance of this work is the fact that differential membrane interactions for metal mixtures versus single metal were observed for toxic ions as a function of lipid structure.

## 2. Results

Structural differences between PA and PS will determine the ion-lipid interactions. The phosphate headgroup of PA is small and readily accessible to ions and, under our experimental conditions, carries a net charge of −1.2 [29,30]. In contrast, PS caries a zwitterionic serine head group linked with a phosphate group to the glycerol backbone and has an overall negative charge of −1 [31]. Large unilamellar liposomes were used as model systems to avoid highly curved liposomes that promote binding events [32].

Metals and lipids were used at equimolar concentrations and the total metal concentration in mixtures was kept the same. All three metals predominantly form divalent cations under the experimental conditions used (Table S1), which allows direct comparison of the results.

### 2.1. Phosphatidic Acid (PA)

Generalized polarization (GP) measurements were carried out at 37 °C, and the reading of 0.058 suggests a fluid membrane, consistent with a reported phase transition temperature from gel to liquid crystalline at 28 °C [20].

The addition of 50 and 100 µM Mn resulted in significant rigidification reflected by higher GP values, whereas similar additions of both 50 and 100 µM Ca or Mg only showed a limited increase for Mg (Table 1).

Next, 1:1 mixtures combining 50 µM of each ion were analyzed in terms of ΔGP, the difference between the respective sample and the control. The increase of rigidity followed the order Ca:Mn > Mg:Mn > Ca:Mg with 2.4×, 1.9× and 1.4× over controls. A comparison to 50 µM metal values suggest that Mn plays a dominant role over Ca and Mg (Table 1). Next, a potential impact of the order of metal additions was assessed. An initial addition of 50 µM Ca was incubated for 5 min before 50 µM Mn was added. The ΔGP readings for Mn containing systems were slightly lower than the binary counterparts, while the change for Ca:Mg was statistically lower than binary values. The order of addition played a minor role and confirmed that POPA is not a relevant binding target for the essential ions Ca and Mg

Binding constants for these ions to POPA have been published [33,34], and these values were used for a more detailed comparison of the binding affinities of two metals at a time.

Equations (1) and (3) show the formation of metal bound to lipid [M_1_L] and [M_2_L]. Equations (2) and (4) show the relationship between the binding constants K_M1_ and K_M2_ with the concentrations of metal [M_1_] and [M_2_] and lipid [L].

(1) $$M_{1}+L⇄M_{1}L$$

(2) $$K_{M_{1}}=\frac{[M_{1}]\left[L\right]}{\left[M_{1}L\right]}$$

(3) $$M_{2}+L⇄M_{2}L$$

(4) $$K_{M_{2}}=\frac{[M_{2}]\left[L\right]}{\left[M_{2}L\right]}$$

The total initial metal concentrations [M_1_]_0_ and [M_2_]_0_ is expressed in Equations (5) and (6) as the sum of free metal and lipid bound metal, whereas the initial lipid concentration [L]_0_ is the sum of free lipid [L] and the lipid fractions that have metals bound in Equation (7).

(5) $${\left[M_{1}\right]}_{0}=[M_{1}]+[M_{1}L]$$

(6) $${\left[M_{2}\right]}_{0}=[M_{2}]+[M_{2}L]$$

(7) $${\left[L\right]}_{0}=\left[L\right]+\left[M_{1}L\right]+\left[M_{2}L\right]$$

[M_1_] can be expressed from Equation (5) and inserted into Equation (2) to rearrange for K_M1_. Next, [M_1_L] can be derived as shown in Equation (8).

$$K_{M_{1}}=\frac{[M_{1}]\left[L\right]}{\left[M_{1}L\right]}=\frac{\left({\left[M_{1}\right]}_{0}-\left[M_{1}L\right]\right)\left[L\right]}{\left[M_{1}L\right]}$$

(8) $$\left[M_{1}L\right]=\frac{\left[L\right]{\left[M_{1}\right]}_{0}}{K_{M_{1}}+\left[L\right]}$$

In an analogy for Equations (3) and (6), [M_2_L] can be expressed (Equation (9))

(9) $$\left[M_{2}L\right]=\frac{\left[L\right]{\left[M_{2}\right]}_{0}}{K_{M_{2}}+\left[L\right]}$$

The substitution of Equations (8) and (9) into Equation (7) results in a cubic equation (see the Supplemental Materials for details).

$${\mathrm{ax}}^{3}+{\mathrm{bx}}^{2}+\mathrm{cx}+d=0$$

This equation was solved using the published binding constants [35] and our experimental metal and lipid concentrations to get the fraction of bound metal for single metal additions at 50 and 100 µM and binary additions of 100 µM (see Supplementary Materials for more details and Table S2). These data were plotted against the experimental GP changes (ΔGP) (Table S2).

This linear regression showed a strong correlation between bound metal concentration and resulting membrane rigidity.

One additional set of experiments was the sequential addition of 50 µM of one metal with 5 min incubation, followed by 50 µM of the second metal, since we have previously observed significant GP and liposome size differences for Cd and Hg mixtures upon sequential additions [18].

The GP values of the sequential mixtures were significantly lower for the Ca:Mg system when compared to binary results.

**Figure 1:**
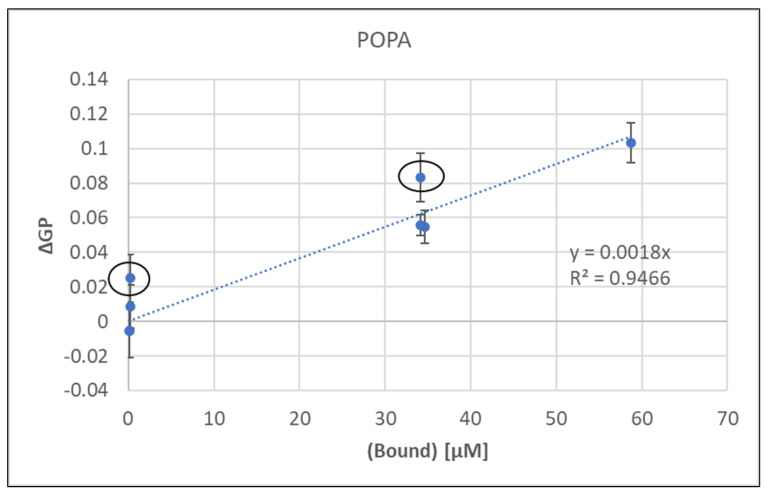
Change in measured GP versus calculated bound metal concentrations of Mn, Ca and Mg and mixtures thereof. Circles represent binary data points that will be discussed in the text.

Since the metal concentrations were the same, the calculated values for bound metal would be identical for sequential and binary mixtures despite the differences in GP. Thus, the linear regression in Figure 1 was used to estimate the bound concentrations for sequential additions based on the observed correlation between bound metal and induced rigidity and these values are shown in Table 2.

While Ca:Mn and Mg:Mn were similar to the calculated binary addition (Table S1), Ca:Mg was significantly higher (~25×) for sequential over binary addition. In fact, the points highlighted in Figure 1 with red circles are binary additions and their ΔGP values are well above the trend line suggesting that more metal may be bound in some binary mixtures.

As previously reported for Cd, Co, Ni and Mn, the acyl side chain architecture strongly impacted metal effects, as fully saturated lipids showed enhanced metal induced membrane rigidification [36,37,38].

Thus, a fully saturated PA was investigated next. Experiments with saturated DMPA were carried out at 55 °C above a reported T_m_ of 52 °C [20] to assess metal binding in the physiologically relevant liquid crystalline phase. GP values for the 50 µM additions of single metals were very similar for all three ions (Table 3).

The controls values were lower than for POPA due to the higher temperature required for DMPA and thus only trends can be compared between the two lipids. In contrast to POPA, the additions of 100 µM metals resulted in statistically significant increases of GP and thus more rigid membranes (Table 3).

The Mn readings were much higher than those of Ca and Mg while all binary metal mixtures exhibited comparable ΔGP values. The overall range of GP changes of the Mg:Mn mixture is similar to POPA, whereas the Ca:Mg is higher for DMPA.

In terms of sequential metal additions, ΔGP ranges suggest a reduced impact of Mn and stronger binding of Mg to DMPA when compared to POPA.

These differential ion interactions with POPA and DMPA were further investigated by DLS to measure the impact of metal addition on liposome size. Higher metal concentrations were used to better illustrate the changes (Table 4).

The single addition of metal ions at 100 µM did not result in statistically relevant changes (Table 3), while both 200 µM of either Mn or Mg increased the size. Overall, Ca had the strongest effect. This is comparable to previous results [29,39].

Both Mn containing mixtures increased the liposome size, whereby the high degree in standard deviation for Ca:Mn was due to increased sample turbidity, suggesting at least the onset of aggregation. In contrast, the Ca:Mg mixture had no effect as seen for their 100 µM values.

Finally, results only differed for the sequential additions in Ca:Mn systems.

The metal effects on the liposome size of DMPA membranes were much more pronounced. Single additions of 200 µM of each metal induced extensive increased between the range of 730 to 850 nm, suggesting the potential of liposome aggregation and increased turbidity of the solutions (Table 4).

Interestingly, 1:1 mixtures with the same total metal concentrations did not induce these large increases. Ca:Mn was similar to 100 µM Ca, suggesting a stronger role. In contrast, the Mg:Mn was close to 100 µM Mn, suggest that Mn dominated the latter interaction.

Sequential additions of Ca and Mn showed no significant size differences compared to binary, whereas Ca and Mg mixtures induced smaller changes (Table 4).

As seen for GP, metal mixtures also behaved very differently compared to single ion additions in terms of liposome size changes.

**Figure 2:**
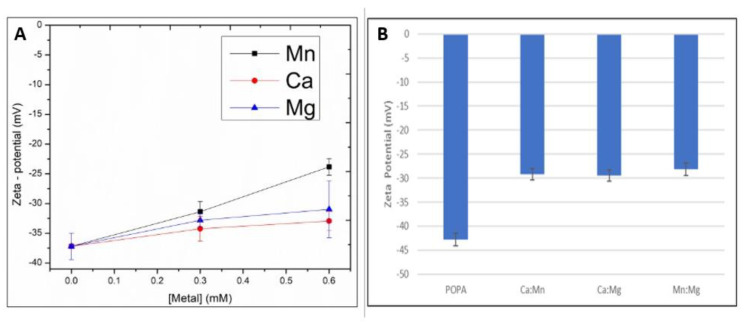
Zeta potential values of POPA LUVs with (**A**) the dose-dependent addition of Mn, Ca and Mg and (**B**) mixtures of Mn, Ca and Mg present in a 1:1 metal mixture to a total metal concentration of 0.3 mM. Samples were measured at 25 °C. Data are obtained as an average of three replicates ± standard deviation (*n* = 3).

Next, changes in zeta–potential upon metal additions were determined. The observed negative zeta potential of pure POPA is expected for negatively charged liposomes (Figure 2). The addition of each metal increased the zeta potential due to screening of negative surface charges. (Figure 2A). At increasing metal concentration (2:1 metal-lipid), the effect of Mn was stronger than Ca and Mg. All metal mixtures induced statistically relevant increases in the zeta potential (Figure 2B). These trends are comparable to GP results in Table 3. The observed changes in zeta potential confirm metal binding to the liposomes, without showing statistically significant differences between the metal mixtures.

### 2.2. Phosphatidylserine (PS)

PS lipids provided two potential ion binding sites, the phosphate and carboxyl groups. Structural studies using NMR demonstrated that the carboxyl group was readily accessible for cation binding due to the head group tilt of PS lipids providing a cavity for metal ions to bind [36,37].

Measurements were carried out at 37 °C in the liquid crystalline phase, reflected in the low GP values of the control. This temperature is well above the phase transition temperature of 14 °C [38].

POPS was significantly rigidified by Mn and Ca (3.4- and 1.5-fold increase), while smaller changes for Mg suggested less binding (Table 5).

The 1:1 mixtures combining 50 µM of each ion exhibited significant GP increases in the order Ca:Mn > Mg:Mn > Ca:Mg with 3.8X, 2.9X and 2X. The impact of Mg was much higher compared to POPA results. In terms of sequential addition; only the ΔGP readings Ca:Mg were statistically lower than binary values. Overall, the order of addition played a minor role. POPS is a stronger binding target for Ca and Mg compared to POPA.

**Figure 3:**
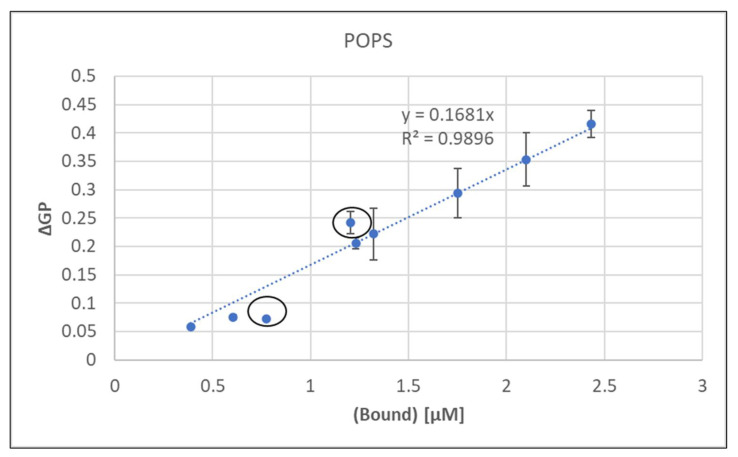
GP versus bound metal concentrations of Mn, Ca, Mg and binary mixtures. The highlighted points will be discussed in the text.

As described for POPA, the degree of metal bound were calculated as outlined above using published binding constants [34,40] (see Table S3). These calculated values were plotted against the experimental GP data in Figure 3, which showed a very good correlation between bound metal and induced membrane rigidity.

As discussed for POPA, calculated values for binary and sequential additions of metal would be identical, although their ΔGP values clearly differ (Table 6). Thus, the equation for the linear regression in Figure 3 was used to calculate bound metal concentrations for sequential additions (Table 6).

Unlike POPS, all three metals caused significant membrane rigidification of the fully saturated DMPS, with the strongest effect for Mn, followed by Ca and Mg (Table 7. Experiments were carried out at 50 °C, in the liquid crystalline phase and well above the T_m_ of 35 °C [41], Metal concentrations had to be reduced to 200 µM to avoid liposome aggregation. Thus, stronger effects were observed at much lower metal concentrations than those used for POPS.

GP values for 200 μM Mn and Ca (Table 8) were similar to the effect of 1000 μM on POPS (Table 6). The most striking contrast to POPS is seen for Mg, with a ΔGP of 0.136 over 0.072 for POPS, although the latter was exposed to a 5× higher metal concentration (Table 6 and Table 8). Mg values were similar to Mn values.

The Mn containing binary mixtures exhibited comparable ΔGP values that were similar to single Mn or Mg additions. Finally, Ca:Mg was lower than the combined values for 100 μM of Ca and Mg. These data again demonstrated that the impact of some metal mixtures clearly deviated from single metal additions.

Sequential additions of the metals did not show a dependence on order for Ca:Mn, while Mg:Mn data were lower than the binary data. Most strikingly was the significant increase of GP for the sequential addition of Ca:Mg over the binary addition. Overall, the impact of Mg was significantly increased for DMPS compared to POPS. These results confirmed that single addition data could not be extrapolated to binary mixtures and in some cases, stepwise additions were different as well.

Next, changes in liposome size were determined by DLS (Table 8). Control liposomes for POPS were 104.8 ± 0.7 nm with a PDI of 0.18. Again, higher metal concentrations were used to better illustrate the effects. The addition of 500 µM metal only showed minor increases, but 1000 µM Mn and Ca doubled the liposome diameter. Indeed, Ca has been shown to induce fusion in PS liposomes [28]. A doubling of the surface area of the POPS liposomes after a fusion event would result in a calculated diameter of 156 nm. Thus, Mn and Ca data suggested membrane fusion.

Alike the Laurdan GP results, Mg only induced a minor change of the hydrodynamic diameter and its effects at 500 μM were much less than size increases for Mg and POPA at 100 and 200 μM (Table 4).

Remarkably, for sequential additions, Mn containing mixtures induced larger size changes compared to binary (Table 9). A more pronounced role for the order of addition was seen for Ca:Mg where Ca(1):Mg(2) induced larger liposomes in the fusion size range, whereas Mg(1):Ca(2) results were similar to the binary system.

In contrast, binary and sequential additions of Ca and Mg did not induce significant size changes in POPA (Table 4).

The addition of the metals to DMPS liposomes resulted in extensive aggregation at concentrations above 200 µM and lower metal concentrations compared to POPS had to be used.

All three metals at 100 and 200 µM resulted in moderate and comparable size increases (Table 8) that were much smaller than seen for DMPA at the same metal concentrations that exhibited up to ~6-fold size increases (Table 4).

Metal mixtures induced moderate changes that were similar for all systems and well below results for DMPA with 2–2.5-fold size increases (Table 4).

For sequential additions, Ca:Mn mixtures were higher than the binary, especially Ca(1):Mn(2) (Table 9).

**Figure 4:**
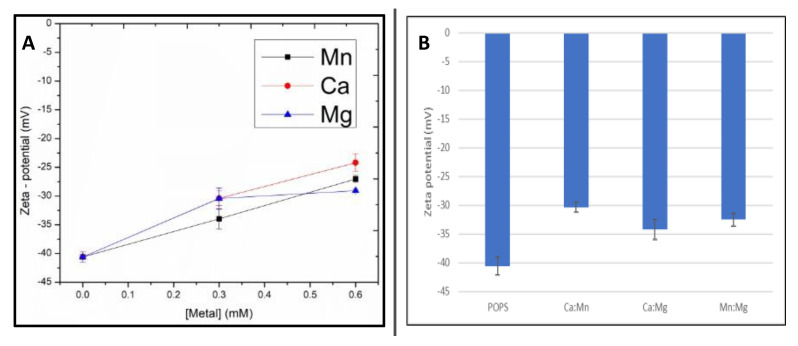
Zeta potential values of POPS LUVs with (**A**) mixtures of Mn, Ca and Mg present in a 1:1 metal mixture to a total metal concentration of 0.3 mM and (**B**) dose-dependent additions of Mn, Ca and Mg. Samples were measured at 25 °C Data are obtained as an average of three replicates ± standard deviation (*n* = 3).

Alike POPA membranes, the zeta potential for POPS was determined and control POPS liposomes had a highly negative zeta potential, as expected for negatively charged lipids (Figure 4).

All metal mixtures induced statistically relevant increases in the zeta potential, whereby, at a 1:1 ratio, Ca and Mg were the strongest (1.7-fold increase). Mn was slightly weaker, in contrast to being the strongest with POPA (Figure 2). At increasing metal concentrations (2:1 metal:lipid), Ca maintained the strongest role, followed by Mn and a slightly reduced effect of Mg. The observed changes in the zeta potential confirmed metal binding to liposomes without showing statistically significant differences between the metals.

Binary additions resulted in the following order Ca:Mn > Mg:Mn and Ca:Mg, with changes within the standard deviations. These trends agreed with the GP trends in Table 4.

## 3. Discussion

We have extensively utilized biomimetic model and complex biological extracts to investigate the membrane interaction of divalent cations such as Cd, Hg, Co, Ni and Mn [29,44,45,46] as a function of lipid composition. Important factors such as metal speciation as well as unspecific binding to buffers are often not considered but significantly affect these interactions (for a review, see [47]). Overall, these interactions induced membrane rigidification and increased in liposome size as reported here. Metal membrane interactions are driven by electrostatic attractions but other factors, such as the chemical characteristics of these metals and the lipid architecture also contribute as outlined below.

Many studies are undertaken with single metal ions while living organism are exposed to metal mixtures. In previous work we had observed for toxic metal mixtures that the order of addition was relevant as the extent of liposome aggregation differed when Cd was added first followed by Hg or vice versa changed [46] and was the rationale for the sequential additions presented here.

The GP data in Table 1 and Table 3 show that Mn had the strongest membrane rigidification on both POPA and DMPA liposomes, whereas Ca and Mg had less effect on POPA but increased more rigidity in DMPA, with a more pronounced increase for Mg.

Finally, sequential additions to POPA were lower than their binary counterparts, especially for Ca:Mg systems, but overall, the order of addition was not important for metal interactions with PAs.

With respect to POPA liposome size, single metals and binary Mn-containing systems induced moderate size increases, whereas the Ca:Mg induced no significant size changes.

In contrast, saturated DMPA liposomes were increased up to six-fold by single ions additions, while binary and, especially, sequential were much lower.

POPA and DMPA differ in their molecular area, with 48.5 Å^2^ versus 43.3 Å^2^ [48,49]. A smaller area and increased surface charge density may facilitate metal complexation and explain the strong preference of Ca, Mn and Mg for DMPA over POPA.

Molecular dynamics simulation revealed that divalent cation reduced the membrane surface charge, which decreased the local proton concentration, leading to a net charge of −2 [50]. PA lipids present a differential binding target for Mn, Ca and Mg individually and in the form of binary metal mixtures, which may result in different impacts in terms of membrane rigidification and surface charge.

### Metal Interactions with PS Lipids

Comparable to PA lipids, Mn had the strongest effect on both POPS and DMPS, followed by Ca, which was stronger than Mg in its interactions in contrast to comparable effects for both ions on PAs. Binary and sequential additions induced higher GP.

Metals induced much larger size increases in POPS, and diameters above 150 nm suggest membrane fusion, as reported previously for Ca [39]. Such as for GP, Mn had the strongest impact. Most sequential additions showed higher values than binary results, emphasizing that, in some cases, the order of addition is important. DMPS showed similar trends with larger increases for sequential over binary as well, again indicating a role for the order of ion addition.

POPS and DMPS differ in their molecular area as well, with 55.0 Å^2^ versus 50.0 Å^2^ [51,52] resulting in and smaller area and increased surface charge density may facilitate metal complexation and explain the strong preference of Ca, Mn and Mg for DMPA over POPA.

Both PA and PS were attractive targets for Mn, which exuded a relatively stronger impact on membrane fluidity and liposome size, compared to Ca and Mg. Mn binding to anionic phospholipid membranes has been reported, resulting in increased membrane rigidity, permeability and aggregation [17,29,51]. Moreover, Mn interaction with POPA and DMPA revealed strong membrane rigidification, liposome size increases and shifts in T_m_ [29].

Ca has also been known for strong interaction with anionic phospholipids and, to a lesser extent, Mg [51]. Ca–PS interactions have been investigated thoroughly [30]. Commonly reported consequences included the formation of PS microdomains with the subsequent ordering of the PS head group [52] and liposomes fusion at high millimolar Ca concentrations. Mg exhibited a weak affinity for both PA and PS, with less impact on the latter, and has been much less characterized in the literature compared to Ca and Mn.

Both experimental and molecular dynamics data documented specific metal-induced changes to membranes in terms of lateral diffusion, membrane thickness and head group tilt [53,54]. Although much less is known about the structural properties of Mn–lipid membranes, Ca and Mg have been studied extensively. A combination of MD simulations and X-ray diffraction studies on DMPC and DPPC showed that Ca and Mg bound differently to the interfacial region of the bilayer [53,55]. While Ca penetrated close to the glycerol backbone, Mg remained at the outer regions of the lipid head group [56] These differences were explained by the higher level of hydration of Mg, which binds its hydration shell much stronger than Ca. Thus, Ca can form dipole–dipole interactions with the phosphate and carbonyl groups of the lipids.

The key characteristics of these metals are compared in Table 9. Mn is chemically distinct from Ca and Mg and, as a first-row transition element, possesses a larger hydrated radius and can adapt more complex coordination chemistry (Table 1). Their relatively low and comparable values eliminate electronegativity as an essential distinction between the ions. Ca and Mg are considered hard, while Mn is a borderline Lewis acid [57,58]. The phosphate and carboxyl groups of PA and PS, categorized as hard ligands, should be attractive for Ca and Mg, whereas Mn targets also include amino and thiol groups [58]. The weak affinity of Mg for both PA and PS does not match these criteria, and Lewis acidity is not a likely explanation for the observed results.

The hydrated radii decrease in order Mn > Mg > Ca [59], and the larger radius combined with the multiple options to adopt chelation geometry support the observed stronger interactions of Mn.

The highest solvation and energy [42] reflecting the tightly bound water of Mg could reduce the affinity for the phosphate and carboxylate groups in PA and PS. Indeed, Bradley et al. reported a weak nanocluster formation by Mg in phosphoinositide containing membranes in contrast to much stronger Ca effects [60], as Ca was able to readily de-solvate, which allowed the formation of coordinated bonds with the phosphoinositide head group [61,62]. In addition, PA–Ca pairs in monolayers of DPPC:DPPA led to strong phase separation, whereas the Mg effects were again much weaker [63].

The data illustrate that more work needs to be done on metal mixtures, as illustrated by one example. It has been reported that Ca and Mg influenced Mn absorption in the GI tract, whereby Ca was shown to outcompete Mn but not Mg if Mn was treated with a much higher concentration of Ca or Mg [64].

## 4. Materials and Methods

Liposome preparation. Liposomes were made by weighing lyophilized powders of the following lipids: 1-palmitoyl-2-oleoyl-*sn*-glycero-3-phosphocholine (POPC), 1,2-dimyristoyl-*sn*-glycero-3-phosphocholine (DMPC), 1-palmitoyl-2-oleoyl-*sn*-glycero-3-phosphate (POPA), 1,2-dimyristoyl-*sn*-glycero-3-phosphate (DMPA), 1-palmitoyl-2-oleoyl-*sn*-glycero-3-phospho-*L*-serine (POPS) and 1,2-dimyristoyl-*sn*-glycero-3-phospho-*L*-serine (DMPS) (Avanti Polar lipids, Alabaster, AL, USA). These lipids were dissolved in 7:3 (*v/v*) chloroform:methanol, purged under argon gas and dried in a vacuum oven overnight to generate a dry lipid film. The films were rehydrated in 20 mM HEPES, 100 mM NaCl (pH 7.4) buffer, which was then agitated by means of vortex, sonication and freeze/thaw cycles, that resulted in the formation of multilamellar vesicles (MLVs). The MLVs underwent extrusion through a polycarbonate filter of 100 nm in pore size, using a mini-extruder (Avanti Polar lipids, Alabaster, AL, USA). This resulted in large unilamellar vesicles (LUVs) of approximately 100 nm in hydrodynamic diameter that was used for fluorescence, light scattering, and zeta potential measurements.

Fluorescence spectroscopy. Fluorescence measurements were used to measure metal-induced changes in membrane fluidity. The solvent sensitive dye, 1-[6-(Dimethylamino) naphthalen-2-yl]dodecan-1-one (Laurdan), was added in a 1:500 dye: lipid molar ratio. Laurdan is sensitive to the polarity of its microenvironment within membranes and experiences a red shift as lipids undergo a phase transition from the gel phase (L_β_) to liquid crystalline phase (L_α_) [65]. The shift in its fluorescence emission is quantified using the parameter, generalized polarization (GP).

$$\mathrm{GP}=\frac{I_{440}-I_{490}}{I_{440}+I_{490}}$$

Laurdan GP measurements were performed using a Cary Eclipse fluorimeter (Agilent technologies, Santa Clara, CA, USA) at an excitation wavelength of 340 nm, and emission at both 440 and 490 nm as an average of 3 measurements and an excitation and emission band pass of 5 nm. Samples, from 3 independent preparations, consisted of 0.1 mM LUVs in small volume quartz cuvettes (Starna Scientific Ltd., Atascadero, CA, USA) with 100–1000 µM MnCl_2_, CaCl_2_, and MgCl_2_. Metals were added to the LUVs and allowed to incubate for 5 min at room temperature prior to any measurements. Temperature was controlled to ±0.1 °C using a circulating water bath (Agilent technologies, Santa Clara, CA, USA). Measurements were conducted at a temperature range between 10–60 °C, dependent on the T_m_ of each lipid system. Samples were equilibrated for at least 1 min/1 °C change in temperature.

Dynamic light scattering (DLS). DLS measurements report changes in the hydrodynamic diameter of nanoparticles up to sub–micron size. Liposome size measurements were conducted using a Zetasizer Nano ZSP (Malvern Instruments, Worcestershire, UK). Samples were measured in triplicate at 37 and 50 °C, which represents the liquid crystalline phase of the lipid systems tested and determine the mean diameter liposome population. 0.1 mM LUVs were measured with and without the increasing concentration of MnCl_2_, CaCl_2_ and MgCl_2_ to assess any metal induced changes. Furthermore, the size distribution of liposomes was narrow as indicated by a polydispersity index (PDI) of less than 0.10 (<10%) for each measurement.

Zeta potential. Zeta potential measurements were conducted to assess the binding affinity of the metals with the PA and PS lipids tested to better explain the changes observed with Laurdan GP, DLS. These were measured using zeta potential folded capillary cells (Zeta sizer nano series DTS 1070, Worcestershire, UK) with 0.3 mM lipid in 20 mM HEPES, 100 mM NaCl (pH 7.4). Metal concentrations were tested in the range of 0.15–0.6 mM, which represent a metal to lipid ratios of up to 2. Three measurements were conducted for each system, and each sample was scanned 50 times per run. Moreover, the Smoluchowski approximation was used to determine the zeta potential of the liposomes in the absence and presence of the metals.

## 5. Conclusions

Changes in membrane rigidification determined by GP followed the trend Mn > Ca > Mg for both PA and PS systems. Mn also showed stronger effects on membrane fluidity and liposome size for both, binary and sequential additions, whereas Ca outcompeted Mg in their mixtures.

Experimental values for binary mixtures were consistently less than the sum of their single metal additions for PA, while the opposite was seen for PS. The order of metal additions was mainly important for mixtures of Ca and Mg.

DLS results showed that Mn had induced the strongest increases in hydrodynamic diameter for DMPS and induced aggregation for DMPA, while Ca had the strongest effects on POPA and POPS. Thus, metal induced size changes showed a distinct dependence on the lipid side chain architecture.

This was confirmed with metal mixtures, as binary effects were generally higher for POPA and POPS but lower for DMPA and DMPS when compared to sequential additions. These differences between single metal, binary and sequential additions and their dependence on the lipid headgroup and side chain architecture are the most striking outcome of this work with biologically relevant implications.

While the detailed characterization of the observed differential effects will require more work, the fact that their impact changes in metal mixtures and with the lipid composition is interesting implying that metal effects could vary locally within a cell. Moreover, while the fusogenic property of Ca is well studied, both in vitro and in vivo [19,62], these data suggest that other metals may modulate and enhance or reduce Ca induced fusion.

Thus, the dose and spatiotemporal-dependent effects of metal mixtures—in this case, nontoxic and essential metals—will have to be considered to better understand their physiological role. The strong dependence on lipid head group and side chain structure allows differential localized effects within a cell, between cell types and upon metabolically induced changes in lipid composition. These consequences and others reported metal effects in the literature, such as PA charge changes and altered membrane domain formation for phosphoinositides, may have profound effects on proper cellular functions [17].

## Figures and Tables

**Table 1:** Tabulated GP and ΔGP values of POPA lipid with varying concentration of Mn, Ca, Mg and metal mixtures.

	GP		ΔGP
Pure POPA	0.058 ± 0.007	Ca:Mn	0.083 ± 0.007
50 µM Mn	0.11 ± 0.01	Mn:Mg	0.054 ± 0.001
100 µM Mn	0.16 ± 0.01	Ca:Mg	0.024 ± 0.002
50 µM Ca	0.05 ± 0.02	Ca(1):Mn(2)	0.062 ± 0.008
100 µM Ca	0.07 ± 0.01	Mn(1):Ca(2)	0.057 ± 0.004
50 µM Mg	0.05 ± 0.01	Mn(1):Mg(2)	0.064 ± 0.005
100 µM Mg	0.08 ± 0.01	Mg(1):Mn(2)	0.056 ± 0.007
		Ca(1):Mg(2)	0.011 ± 0.005
		Mg(1):Ca(2)	0.011 ± 0.006

**Table 2:** Estimated bound metal fraction for sequential additions using Figure 1.

Order of Addition	ΔGP	[Bound] (µM)
Ca(1):Mn(2)	0.06	34.29
Mn(1):Ca(2)	0.06	31.86
Mn(1):Mg(2)	0.06	35.56
Mg(1):Mn(2)	0.06	31.08
Ca(1):Mg(2)	0.01	6.16
Mg(1):Ca(2)	0.01	5.93

**Table 3:** Tabulated GP and ΔGP values of DMPA lipid with Mn, Ca, Mg and metal mixtures.

	GP		ΔGP
Pure DMPA	0.055 ± 0.004	Ca:Mn	0.043 ± 0.001
50 µM Mn	0.070 ± 0.007	Mn:Mg	0.05 ± 0.01
100 µM Mn	0.22 ± 0.03	Ca:Mg	0.04 ± 0.02
50 µM Ca	0.07 ± 0.01	Ca(1):Mn(2)	0.06 ± 0.01
100 µM Ca	0.106 ± 0.001	Mn(1):Ca(2)	0.047 ± 0.004
50 µM Mg	0.082 ± 0.005	Mn(1):Mg(2)	0.05 ± 0.01
100 µM Mg	0.106 ± 0.003	Mg(1):Mn(2)	0.050 ± 0.001
		Ca(1):Mg(2)	0.057 ± 0.008
		Mg(1):Ca(2)	0.03 ± 0.01

**Table 4:** Tabulated hydrodynamic diameter of POPA and DMPA with Mn, Ca, Mg and metal mixtures.

	POPA	DMPA
	Hydrodynamic Diameter (nm)	Hydrodynamic Diameter (nm)
pure lipid	118.8 ± 0.5	137.7 ± 1.5
100 µM Mn	123.6 ± 1.2	300.3 ± 16.3
200 µM Mn	144.7 ± 4.9	736.7 ± 51.0
100 µM Ca	107.0 ± 2.8	222.8 ± 24.9
200 µM Ca	169.0 ± 6.5	817.1 ± 19.5
100 µM Mg	122.5 ± 1.1	193.2 ± 8.9
200 µM Mg	144.6 ± 6.4	849.9 ± 6.4
Ca:Mn	154.7 ± 25.9	224.8 ± 27.5
Mn:Mg	144.5 ± 2.0	340.1 ± 22.9
Ca:Mg	117.2 ± 0.6	229.2 ± 30.8
Ca(1):Mn(2)	131.8 ± 12.6	197.9 ± 3.3
Mn(1):Ca(2)	165.5 ± 11.2	181.5 ± 9.1
Mn(1):Mg(2)	142.2 ± 2.6	251.2 ± 27.0
Mg(1):Mn(2)	129.6 ± 10.3	150.8 ± 3.6
Ca(1):Mg(2)	108.4 ± 1.5	184.6 ± 1.0
Mg(1):Ca(2)	111.9 ± 1.4	199.4 ± 18.7

**Table 5:** Tabulated GP and ΔGP values of POPS with varying concentration of Mn, Ca, Mg and metal mixtures.

	GP		ΔGP
Pure POPS	−0.092 ± 0.004	Ca:Mn	0.35 ± 0.04
500 µM Mn	0.113 ± 0.009	Mn:Mg	0.29 ± 0.04
1000 µM Mn	0.32 ± 0.02	Ca:Mg	0.22 ± 0.04
500 µM Ca	−0.017 ± 0.004	Ca(1):Mn(2)	0.39 ± 0.03
1000 µM Ca	0.15 ± 0.02	Mn(1):Ca(2)	0.37 ± 0.02
500 µM Mg	−0.034 ± 0.001	Mn(1):Mg(2)	0.24 ± 0.06
1000 µM Mg	−0.020 ± 0.003	Mg(1):Mn(2)	0.29 ± 0.03
		Ca(1):Mg(2)	0.075 ± 0.001
		Mg(1):Ca(2)	0.098 ± 0.001

**Table 6:** Estimation of bound metal for sequential additions based on Figure 3.

Order of Addition	ΔGP	[Bound] (µM)
Ca(1):Mn(2)	0.39	2.31
Mn(1):Ca(2)	0.37	2.18
Mn(1):Mg(2)	0.24	1.43
Mg(1):Mn(2)	0.29	1.73
Ca(1):Mg(2)	0.08	0.45
Mg(1):Ca(2)	0.10	0.59

These values were comparable to the calculated binary values (Table S2) for Mn containing mixtures but ~2.5× lower for Ca:Mg with a higher ΔGP for Mg(1):Ca(2).

**Table 7:** Tabulated GP and ΔGP values of DMPS with varying concentration of Mn, Ca, Mg and metal mixtures.

	GP		ΔGP
Pure DMPS	−0.086 ± 0.009	Ca:Mn	0.35 ± 0.03
100 µM Mn	0.30 ± 0.07	Mn:Mg	0.40 ± 0.03
200 µM Mn	0.34 ± 0.03	Ca:Mg	0.229 ± 0.004
100 µM Ca	0.11 ± 0.01	Ca(1):Mn(2)	0.36 ± 0.02
200 µM Ca	0.16 ± 0.05	Mn(1):Ca(2)	0.430 ± 0.007
100 µM Mg	0.035 ± 0.001	Mn(1):Mg(2)	0.25 ± 0.01
200 µM Mg	0.05 ± 0.02	Mg(1):Mn(2)	0.239 ± 0.008
		Ca(1):Mg(2)	0.42 + 0.02
		Mg(1):Ca(2)	0.39 ± 0.03

**Table 8:** Tabulated hydrodynamic diameter of POPS and DMPS lipid with Mn, Ca, Mg and metal mixture.

	POPS		DMPS
	Hydrodynamic Diameter (nm)		Hydrodynamic Diameter (nm)
Pure POPS	104.8 ± 0.7	Pure DMPS	110.5 ± 1.2
500 µM Mn	106.5 ± 0.9	100 µM Mn	127.4 ± 2.2
1000 µM Mn	201.3 ± 3.7	200 µM Mn	139.0 ± 14.4
500 µM Ca	109.9 ± 1.2	100 µM Ca	121.7 ± 1.2
1000 µM Ca	225.9 ± 2.7	200 µM Ca	134.8 ± 3.2
500 µM Mg	110.0 ± 4.4	100 µM Mg	123.6 ± 1.9
1000 µM Mg	109.0 ± 0.5	200 µM Mg	130.8 ± 3.7
Ca:Mn	165.8 ± 0.7	Ca:Mn	126.7 ± 2.1
Mn:Mg	152.6 ± 0.9	Mn:Mg	126.6 ± 0.9
Ca:Mg	130.4 ± 1.7	Ca:Mg	124.1 ± 3.5
Ca(1):Mn(2)	184.3 ± 2.8	Ca(1):Mn(2)	196.5 ± 11.6
Mn(1):Ca(2)	189.4 ± 2.9	Mn(1):Ca(2)	157.9 ± 13.6
Mn(1):Mg(2)	187.3 ± 0.9	Mn(1):Mg(2)	132.6 ± 6.9
Mg(1):Mn(2)	168.1 ± 0.7	Mg(1):Mn(2)	134.2 ± 3.8
Ca(1):Mg(2)	173.4 ± 1.6	Ca(1):Mg(2)	120.3 ± 3.5
Mg(1):Ca(2)	128.4 ± 2.5	Mg(1):Ca(2)	130.0 ± 0.7

Size changes induced by equimolar binary additions were in the range estimated for membrane fusion (>150 nm), and only Ca:Mg sizes were below. The order of Ca:Mn > Mg:Mn in the binary mixtures was similar for POPA, but Ca:Mg did not affect the size of the POPA liposomes (Table 4), in contrast to POPS.

**Table 9:** Tabulated chemical properties of Ca, Mn and Mg.

	Mn^2+^:	Ca^2+^:	Mg^2+^:
Ionic radii (Ǻ):	0.91	0.99	0.65
Hydrated radii (Ǻ):	4.38	4.19	4.28
Coordination number and geometry	6, Octahedral 5, Trigonal bipyramidal	6, Octahedral	6, Octahedral
Lewis acidity	Borderline	Hard	Hard
Solvation energies ^a^ (kcal/mol)	−416.8	−354.7	−433.3
Electronegativity	1.5	1.0	1.2

^a^ Value based on quasi-chemical approximation [42,43].

## Data Availability

Data was contained within the article or Supplementary Materials.

## Supplementary Materials

The following supporting information can be downloaded at: https://www.mdpi.com/article/10.3390/ijms24021066/s1.
