# Supplementary material for: Lipid Structure Determines the Differential Impact of Single Metal Additions and Binary Mixtures of Manganese, Calcium and Magnesium on Membrane Fluidity and Liposome Size
Source: Int J Mol Sci. 2023 Jan 5;24(2):1066. doi: 10.3390/ijms24021066 (PMC9860990; doi:10.3390/ijms24021066)
Supplement: Supplementary file 1 [file ijms-24-01066-s001.zip › ijms-2114581-supplementary.pdf]

Lipid structure determines the differential impact of single metal additions and binary mixtures of manganese, calcium and magnesium on membrane fluidity and liposome size

Kevin Sule [1], Max Anikovskiy [2], and Elmar J. Prenner [1,*]

[1] *Department of Biological Sciences, University of Calgary, Calgary, AB T2N 1N4, Canada*

[2] *Department of Chemistry, Nanoscience Program, University of Calgary, Calgary, AB T2N 1N4, Canada*

[*] *Corresponding author: eprenner@ucalgary.ca*

Calculation of bound metal based on published binding constants (refs):

Equations 8 and 9 as shown in the manuscript: $[M_1L] = \frac{[L][M_1]_0}{K_{M_1}+[L]}$ (8)

$$[M_2L] = \frac{[L][M_2]_0}{K_{M_2}+[L]} \quad (9)$$

These equations were substituted into equation 7

$$[L]_0 = [L] + [M_1L] + [M_2L] \quad (7)$$

Yielding the following expression:

$$[L]^3 + \left([M_1]_0 + [M_2]_0 + K_{M_1} + K_{M_2} - [L]_0\right)[L]^2 + \left(K_{M_1}K_{M_2} + K_{M_1}[M_2]_0 + K_{M_2}[M_1]_0 - K_{M_1}[L]_0 - K_{M_2}[L]_0\right)[L] - K_{M_1}K_{M_2}[L]_0 = 0 \quad (10)$$

This expression has the format of a cubic equation that was solved as shown below

$$ax^3 + bx^2 + cx + d = 0$$

$$a = 1$$

$$b = [M_1]_0 + [M_2]_0 + K_{M_1} + K_{M_2} - [L]_0$$

$$c = K_{M_1}K_{M_2} + K_{M_1}[M_2]_0 + K_{M_2}[M_1]_0 - K_{M_1}[L]_0 - K_{M_2}[L]_0$$

$$d = -K_{M_1}K_{M_2}[L]_0$$

$$x = y - \frac{b}{3a}$$

$$y^3 + \beta y + \gamma = 0$$

$$\beta = \frac{3ac - b^2}{3a^2}$$

$$\gamma = \frac{2b^3 - 9abc + 27a^2d}{27a^3}$$

$$\Delta = 4\beta^3 + 27\gamma^2$$

$$y_k = 2\sqrt{-\frac{\beta}{3}}cos\left(\frac{1}{3}arccos\left(\frac{3\gamma}{2\beta}\sqrt{-\frac{3}{\beta}}\right) - \frac{2\pi k}{3}\right)$$

The equation was used in excel to calculate the concentrations of metal bound to liposomes for single and binary metal additions to model membranes (Tables S2 and S3).

Table S1. Metal speciation of $CaCl_2$, $MnCl_2$, $MgCl_2$ under the experimental conditions[a] determined through Visual Minteq Software.

**Metal**	**Species name**	**Percent distribution (%)**
Calcium (Ca)	Ca^{2+}	91.698
	$CaCl^+$	8.302
Manganese (Mn)	Mn^{2+}	96.137
	$MnOH^+$	0.029
	$MnCl_2$	0.358
	$MnCl^+$	3.466
Magnesium (Mg)	Mg^{2+}	87.451
	$MgCl^+$	12.545

[a] *20 mM Hepes, 100 mM NaCl (pH 7.4)*

Table S2: Calculated bound metal concentrations for single and binary additions to POPS

[Metal] (μM)		[Bound] (μM)	ΔGP
Mn	50	34.629	0.054668
	100	58.721	0.103218
Ca	50	0.121	-0.00539
	100	0.241	0.008572
Mg	50	0.103	-0.0054
	100	0.205	0.024928
Ca:Mn		34.121	0.08331
Mn:Mg		34.102	0.0055736
Ca:Mg		0.223	0.023775

POPS

Table S3 Calculated bound metal concentrations for single and binary additions to POPS

[Metal] (μM)		[Bound] (μM)	ΔGP
Mn	500	1.231	0.205446
	1000	2.432	0.415722
Ca	500	0.605	0.075248
	1000	1.204	0.242058
Mg	500	0.388	0.059196
	1000	0.774	0.072226
Ca:Mn		2.1017	0.353298
Mn:Mg		1.7508	0.294317
Ca:Mg		1.3210	0.222077
